# Image-based surgical site infection algorithms to support home-based post-cesarean monitoring: Lessons from Rwanda

**DOI:** 10.1371/journal.pgph.0001584

**Published:** 2023-02-13

**Authors:** Barnabas Tobi Alayande, Siona Prasad, Monique Abimpaye, Laban Bakorimana, Anne Niyigena, Jonathan Nkurunziza, Vincent K. Cubaka, Fredrick Kateera, Richard Fletcher, Bethany Hedt-Gauthier

**Affiliations:** 1 Center for Equity in Global Surgery, University of Global Health Equity, Kigali, Rwanda; 2 Program in Global Surgery and Social Change, Harvard Medical School, Boston, Massachusetts, United States of America; 3 Harvard University, Boston, Massachusetts, United States of America; 4 Save the Children, International, London, United Kingdom; 5 Research Department, Partners In Health/Inshuti Mu Buzima, Kigali, Rwanda; 6 Department of Mechanical Engineering, Massachusetts Institute of Technology, Cambridge, Massachusetts, United States of America; 7 Department of Global Health and Social Medicine, Harvard Medical School, Boston, Massachusetts, United States of America; McGill University, CANADA

Surgical site infections (SSIs), which occur in an average of 12.4% (1% to 42%) of women delivering via cesarean in sub-Saharan Africa, are a significant contributor to maternal morbidity and mortality [1]. One key challenge to timely cesarean-associated SSI management is that these infections often develop after hospital discharge, and return to health facilities for follow-up is physically burdensome and financially catastrophic for women [2, 3]. Community Health Workers (CHWs), who already support community-based maternal care in many countries [4], could support home-based post-cesarean monitoring if equipped with tools for accurate SSI diagnosis.

Over the last eight years, our research team has explored several strategies for sustainable, home-based, CHW-supported care for women after cesarean delivery in rural Rwanda. We have demonstrated that home-based follow-up by CHWs is feasible and acceptable [5, 6], but our attempts for simple clinical screenings or sending pictures to general practitioners has resulted in low SSI diagnostic accuracy [5–8]. In contrast, our machine learning image-based diagnostic algorithms are yielding higher accuracy: 81.3% sensitivity and 65.3% specificity for visible image algorithms [9], increasing to 97% sensitivity and 87% specificity when using computer vision techniques and color calibration in the image processing pipeline, and 95% specificity and 84% specificity for thermal image algorithms [10]. We believe these algorithms hold huge potential for remote SSI diagnostics by health workers in the community in low- and middle-income countries (LMICs), and here we offer some lessons learned to improve others’ efforts as they work towards this goal.

The first set of lessons are with regards to *database curation*. To develop machine learning algorithms for SSI diagnosis, we needed sets of wound images and SSI diagnoses, neither of which were straightforward to capture. For our first attempts, CHWs and data collectors captured wound images “free form”, using the cameras on the Samsung Galaxy Tab 3 tablet without any additional guidance. Camera settings and stability affect the resolution of an image, and environmental conditions, including lighting and shadows, affect brightness and contrast of visible images. The resulting images were difficult to work with due to varying orientation, angulation, and brightness and, as a result, required extensive manual editing before we could proceed. Even with applications that standardize image capture prior to the implementation of an algorithm, images require manual image pre-processing to account for variations in lighting conditions, camera position and stability, which may be dependent on health worker training.

In later attempts, we developed a WoundScreener App (Fig 1) with computer vision features that enabled automatic image scaling and rotation correction. This App is not yet available to the public and is being optimized by a technology hub. This App used a bounding box algorithm and a rectangular polygon model to find the outline of the wound frame and automatically cropped the images in a standardized manner, similar to how some modern banks employ a mobile application that uses a smart phone camera to detect the edges of a check for deposit. Our Wound Screener application makes use of a paper frame, known as a computer vision target, that is placed over the wound. This target also includes a color pattern that enables automatic image color calibration; this is particularly important if the ambient lighting is not standardized. The use of color calibration along with computer vision methods improved quality of our image training data and considerably improved the model performance, resulting in an increase in area under the curve (AUC) accuracy from 0.64 to 0.86 (9). Using applications to standardize image capture, such as the WoundScreener App, leads to improved color and size consistency across the images that will facilitate algorithm development and deployment [11], and, in our case, eliminated the need for manual cropping (9).

**Fig 1:**
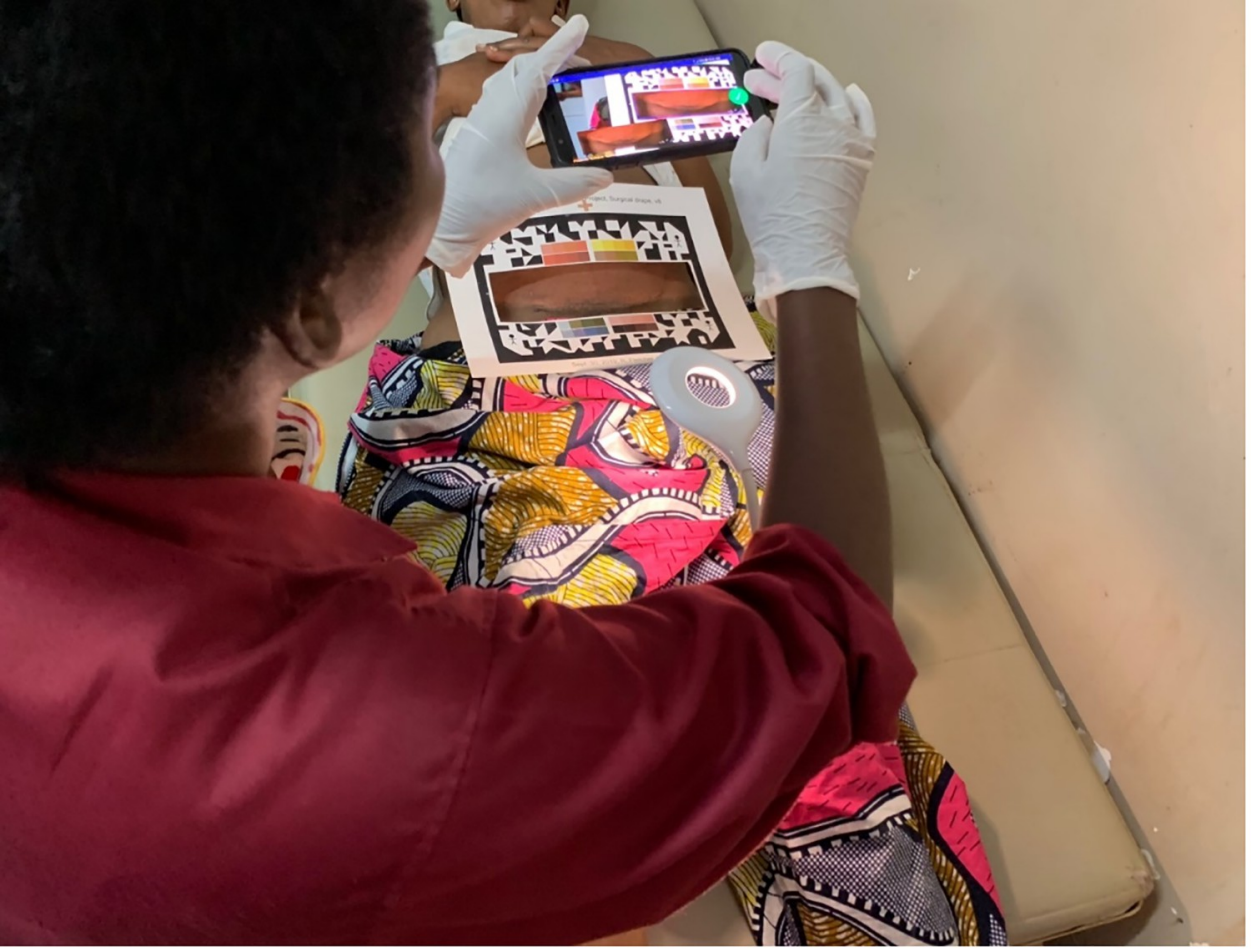
Health provider using the WoundScreener App.

Gold standards for SSI diagnoses are debatable and also challenging to achieve. In addition to clinical examination, confirmative pathology could be used. However, many rural health facilities in LMICs do not have access to bacteriology labs and we found establishing such capacity incredibly challenging [12]. Further, even when available, non-pathogenic bacteria can be misdetected on wound swabs, adding uncertainty to diagnoses [13]. In rural Rwanda, SSI by clinical diagnosis is standard of care and for our algorithm development, we used this clinical diagnosis as the gold standard, as this replicates what the patient would have received if she returned to the facility for wound monitoring. However, if infrastructure and physical and human resources allow, wound pathology or multiple independent physical exams could increase the confidence in the SSI diagnosis that the algorithm is predicting.

Our second set of lessons is with regards to *algorithm development*; the common misconception is that algorithm development flows easily once you have a set of labelled data (e.g., images with a corresponding diagnosis). However, a common challenge in algorithm development is adjusting for unbalanced data which can negatively affect the trained algorithm’s detection of the “minority class” [14]. In our work, we had considerably more labelled data from patients without an SSI; without adjusting the training process, we risked low sensitivity in the resulting algorithm. We addressed these imbalances through the application of Synthetic Minority Oversampling Technique (SMOTE) where we synthetically generated more images in the minority class and modified class weights so that the minority class is assigned a higher weight and misclassification of this group is penalized more through a custom loss function. The class imbalance also influenced our choice of evaluation metrics, where we prioritized AUC, as well as MCC (Matthews Correlation Coefficient) which tracks both false positives and false negatives [15].

Finally, we offer lessons learned with regard to *algorithm generalizability*. Variations in skin tone, image capture method, environmental lighting conditions, smartphone technology, and the small size of our datasets contribute to challenges in algorithm generalizability. Our work was done in Rwanda among women with darker skin tones; given that the color of an infected site can present differently depending on the skin tone of the patient, the algorithm must be evaluated in other populations before it is deployed. We anticipate that the visible algorithm will not generalize broadly and as a result, we are collecting new labelled data from other populations with the goal of developing an algorithm that can work in more places. In addition, we are also exploring the use of thermography, which records the distribution of temperature across the wound surface, and is independent of skin color. Our enthusiasm for thermal image algorithms includes the increased generalizability across populations, a hypothesis we are currently testing.

## Conclusion

Image-based surgical site infection algorithms to support home-based post-cesarean monitoring by community-based health workers should be further explored, but developers must proceed with care. While our work shows great promise, questions concerning contextual standards for SSI diagnosis, standardized image capture for database curation, and technical concerns surrounding algorithm development exist. Image-based algorithms are rarely fully generalizable across contexts, and work must be done acknowledging variation in diagnostic standards, physical environment, and skin tone.
